# NADPH-Oxidase, Rho-Kinase and Autophagy Mediate the (Pro)renin-Induced Pro-Inflammatory Microglial Response and Enhancement of Dopaminergic Neuron Death

**DOI:** 10.3390/antiox10091340

**Published:** 2021-08-25

**Authors:** Andrea Lopez-Lopez, Begoña Villar-Cheda, Aloia Quijano, Pablo Garrido-Gil, María Garcia-Garrote, Carmen Díaz-Ruiz, Ana Muñoz, José L. Labandeira-Garcia

**Affiliations:** 1 Laboratory of Cellular and Molecular Neurobiology of Parkinson’s Disease, Research Center for Molecular Medicine and Chronic Diseases (CIMUS), Department of Morphological Sciences, IDIS, University of Santiago de Compostela, 15782 Santiago de Compostela, Spain; andrealopez.lopez@usc.es (A.L.-L.); bego.villar@usc.es (B.V.-C.); aloia.quijano.ocampo@usc.es (A.Q.); pablo.garrido@usc.es (P.G.-G.); maria.garcia.garrote@usc.es (M.G.-G.); mdelcarmen.diaz@usc.es (C.D.-R.); anamaria.munoz@usc.es (A.M.); 2 Networking Research Center on Neurodegenerative Diseases (CiberNed), 28031 Madrid, Spain

**Keywords:** angiotensin, autophagy, dopamine, microglia, neuroinflammation, neurodegeneration, Parkinson, prorenin, renin, ROCK

## Abstract

Dysregulation of the tissue renin–angiotensin system (RAS) is involved in tissue oxidative and inflammatory responses. Among RAS components, renin, its precursor (pro)renin and its specific receptor (PRR) have been less investigated, particularly in the brain. We previously showed the presence of PRR in neurons and glial cells in the nigrostriatal system of rodents and primates, including humans. Now, we used rat and mouse models and cultures of BV2 and primary microglial cells to study the role of PRR in microglial pro-inflammatory responses. PRR was upregulated in the nigral region, particularly in microglia during the neuroinflammatory response. In the presence of the angiotensin type-1 receptor blocker losartan, to exclude angiotensin-related effects, treatment of microglial cells with (pro)renin induces the expression of microglial pro-inflammatory markers, which is mediated by upregulation of NADPH-oxidase and Rho-kinase activities, downregulation of autophagy and upregulation of inflammasome activity. Conditioned medium from (pro)renin-treated microglia increased dopaminergic cell death relative to medium from non-treated microglia. However, these effects were blocked by pre-treatment of microglia with the Rho-kinase inhibitor fasudil. Activation of microglial PRR enhances the microglial pro-inflammatory response and deleterious effects of microglia on dopaminergic cells, and microglial NADPH-oxidase, Rho-Kinase and autophagy are involved in this process.

## 1. Introduction

The renin–angiotensin system (RAS) is present in most tissues, including the brain, and dysregulation of tissue RAS is involved in the inflammatory response related to different diseases and aging-related pro-inflammatory processes [1,2,3,4]. Recently, the major role of the tissue RAS in the COVID-19 pandemic has also been shown, and Angiotensin-converting enzyme 2 (ACE2), a central component of the RAS, is the entry receptor for SARS-CoV-2 [5,6]. In brief, the RAS is organized into two axes that counteract each other to establish a correct balance in physiological conditions: a pro-inflammatory and pro-oxidative arm mainly represented by angiotensin II (AngII) and angiotensin type 1 (AT1) receptors, and an anti-inflammatory and anti-oxidative arm formed by AngII/AT2 receptors together with Ang1-7/Mas receptors (MasR) [7,8]. ACE2 plays a central role in the balance by transforming components of the pro-inflammatory arm (AngI and particularly AngII) into components of the anti-inflammatory axis (Ang1-9, and particularly Ang1-7) [7,8]. Among RAS components, renin and its precursor (pro)renin (PR) together with their specific receptor (PRR) have been less investigated, despite they play a major role in RAS function. PR and PRR play a double functional role (i.e., AngII dependent and independent functions) [9,10]. First, AngII-dependent actions, as it is classically known that renin hydrolyzes the RAS precursor protein angiotensinogen into AngI (then transformed into AngII by ACE); however, binding of renin to PRR increases the catalytic activity of renin by about 4–5 times, and binding of pro-renin induces catalytic activity similar to that of renin [9,10,11,12]. Second, AngII-independent actions by triggering a PRR-derived intracellular signaling cascade, which effects are usually considered to be pro-inflammatory and included as a component of the pro-oxidative pro-inflammatory arm [9,10,11,12]. However, the PRR intracellular effects are complex and the intracellular pathways involved in these effects are unclear.

In the brain, a number of previous studies have shown that local RAS is involved in neuroinflammation and neurodegeneration, which has been reviewed in detail elsewhere [13,14]. We showed a major role of the local brain RAS in neuroinflammation and dopaminergic degeneration in Parkinson’s disease (PD) models, including classical 6-hydroxydopamine (6-OHDA)- and MPTP-induced neurotoxic PD models in rats and mice, showing that AngII, via AT1 receptors, increases the dopaminergic degeneration process by amplifying the inflammatory response and intraneuronal levels of oxidative stress, and that microglial cells play a major role in this process, which is inhibited by treatment AT1 receptor antagonists [15,16]. However, we also showed the presence of PRR in neurons and glial cells in the nigrostriatal system of rodents and primates, including humans [17,18,19]. Interestingly, it was also observed that in the hypothalamus prorenin induced stimulatory effects on microglial activation and pro-inflammatory cytokine production in the presence of the AT1 antagonist losartan, and that the M1 activated phenotype was induced in retinal microglia exposed to prorenin, which was not affected by the AT1 antagonist candesartan, revealing a direct effect of prorenin [20,21]. Previous studies from our group and others have revealed that RAS regulates microglial responses and that these effects are mediated by NADPH-oxidase, Rho-kinase (ROCK) and other inflammation-related microglial components [7,22,23,24]. However, the role of PRR activation in the pro-inflammatory microglial mechanisms has not been clarified, and the effect of microglial PRR activation in dopaminergic degeneration is unclear. In the present study, we investigated changes in PRR expression in the nigral region of several animal models of neuroinflammation, and we used cultures of microglial cells to study the effects of PR/PRR signaling in microglia.

## 2. Materials and Methods

### 2.1. Experimental Design

**Figure 1:**
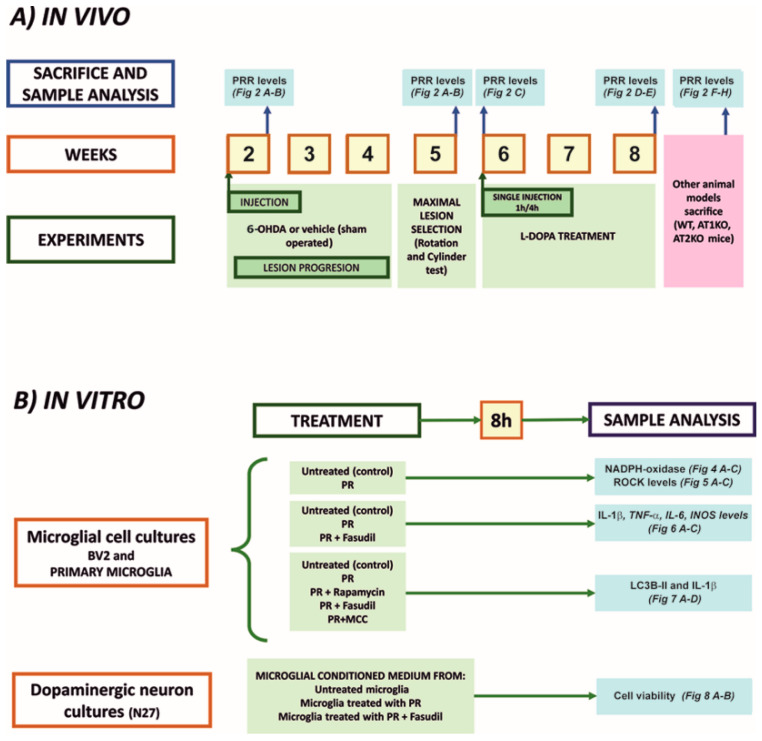
Time course of in vivo (**A**) and in vitro experiments using microglial cell cultures (BV2 and primary microglia) and N27 dopaminergic neuron cell line cultures (**B**). 6-OHDA, 6-hydroxydopamine; IL-1β, interleukin1β; IL-6, interleukin 6; iNOS, inducible Nitric oxide synthases; LC3B, microtubule-associated protein 1 light chain 3 beta; MCC, *N*-[[(1,2,3,5,6,7-Hexahydro-s-indacen-4-yl)amino]carbonyl]-4-(1-hydroxy-1-methylethyl)-2-furansulfonamide; NADPH oxidase, nicotinamide adenine dinucleotide phosphate oxidase; PR, (pro)renin; PRR (pro)renin receptor.

The experimental design is summarized in Figure 1. First, we studied PRR mRNA (RT-PCR) or protein (Western blot) expression in several rodent models (see Section 2.2 for details), which are known to have increased levels of pro-inflammatory markers in the nigral region. Rats were injected in the medial forebrain bundle with the dopaminergic neurotoxin 6-OHDA, and the nigral region was analyzed for PRR expression one week after the injection (i.e., during the acute process of neuroinflammation and neurodegeneration; *n* = 15) or four weeks after the lesion (i.e., when the degenerative process and neuroinflammation had declined; *n* = 15) [25,26]; four weeks after the lesion, a group of rats was injected with L-DOPA to induce dyskinetic behavior, which is accompanied by an increase in pro-inflammatory markers in the nigra [27,28,29], and nigral PRR expression was analyzed. Then, we studied PRR expression in mice deficient for other RAS receptors that are characterized by downregulation of pro-inflammatory markers (AT1 KO mice; *n* = 8) [30,31] or upregulation of pro-inflammatory markers (AT2 KO mice; *n* = 24) [32]. Furthermore, we used adult brains from this last model to isolate microglia (using anti-CD11b microbeads) and neurons (using laser microdissection) to know in which cells took place the observed changes.

In the second set of experiments, we used cultures of the BV2 microglial cell line and cultures of primary microglia to study the effects of activation of PRR with recombinant PR on major components involved in the microglial pro-inflammatory response: NADPH-oxidase complex, RhoA/Rho-kinase pathway, IL-1β, TNF-α, iNOS, autophagic markers, inflammasome markers. Finally, we treated cultures of N27 dopaminergic neurons with conditioned medium from microglia treated or untreated with PR, in the absence or presence of low doses of the dopaminergic neurotoxin 6-OHDA, to know the effect of microglial PRR activation on dopaminergic cell death. All cultures were treated in the absence of any source of angiotensinogen (i.e., serum or astrocytes) to prevent its possible hydrolysis by PR to generate AngI/AngII. In addition, all cultures were treated with the AT1 antagonist losartan to exclude any possible PR-induced pro-inflammatory effect generated by AngII.

### 2.2. Animal Models

Adult female Sprague–Dawley rats (weighing about 250 g; total *n* = 84; obtained from the Galician Center for Animal Experiments, CEBEGA) and male adult mice (weighing 20–25 g; total *n* = 64) were used. Female rats were used because the important increase in body weight of male rats in long-term experiments, such as those analyzing dyskinesias in long-term dopaminergic denervated rats, may affect motor behavioral tests. Mice were WT C57BL-6 mice (Charles River L’Arbresle, France) AT1a KO (Jackson Laboratory Bar Harbor, ME, USA) and AT2 KO mice (generous gift of Dr. Daniel Henrion). All animals were housed under a 12 h light/dark cycle and with ad libitum access to food and water. All experiments were carried out in accordance with the European Communities Council Directive 2010/63/EU and Directive 86/609/EEC and were approved by the corresponding committee of the Regional Government and the University of Santiago de Compostela (protocol 15012/2021/012; last revision 16 April 2021).

PD and dyskinetic models were performed as previously described [29]. Briefly, unilateral lesions of the dopaminergic system were performed by injection into the right medial forebrain bundle of 12 µg of 6-OHDA hydrobromide (Sigma-Aldrich, St Louis, MO, USA) in 4 µL of sterile saline containing 0.2% ascorbic acid (*n* = 30) using a stereotaxic system (Kopf Instruments, Tujunga, CA, USA). The stereotaxic coordinates were 3.7 mm posterior to the bregma, −1.6 mm lateral to the midline, and 8.8 mm ventral to the skull [33]. Rats with maximal dopaminergic denervation were selected using rotation [34] and cylinder tests [35]. Sham-operated rats, injected with vehicle, were used as controls (*n* = 9). Rats were sacrificed one week (short-term lesions) and one month (long-term lesions) after 6-OHDA injection.

A subgroup of rats with long-term lesions was selected for induction of dyskinetic behavior. Before L-DOPA injection, the complete unilateral dopaminergic denervation of these rats was confirmed using the cylinder test and the amphetamine-induced rotational test (2.5 mg/kg). Rats were then treated with L-DOPA methyl ester (12 mg/kg; Sigma) plus benserazide (10 mg/kg; Sigma) (*n* = 24) or saline (*n* = 21) and sacrificed 1hour or 4 h after a first L-DOPA injection or received a daily injection of L-DOPA for 3 weeks to induce more chronic dyskinetic behavior and sacrificed 90 min after the last injection. Quantification of dyskinesia was performed according to [29,36].

Further details on Section 2.2 are included as Supplementary Methods and Supplementary Figure S1.

### 2.3. Isolation of Microglia from Adult Brains

The microglia isolation was performed as previously described [32]. Briefly AT2 KO (*n* = 8) and WT (*n* = 8) mouse brains were removed and homogenized in the presence of papain (1 mg/mL; Sigma), dispase (6 U/mL; Sigma) and DNase (20 U/mL; Sigma). After digestion, cell suspension was washed and centrifuged in Percoll (Sigma) to remove myelin. The pellet, containing cells, was washed, filtered, and centrifuged at 400 g for 5 min. Then cell suspension was incubated with the Anti-CD11b Microbeads (130-093-634, MACS Miltenyi Biotec Auburn, CA, USA). The cell-bead suspension was washed and applied onto MS columns (MACS Miltenyi Biotec) according to manufacturer instructions. Microglial cells were washed, resuspended on lysis buffer (RNeasy Microkit, Qiagen, Germany) and stored at −80 °C until further RNA extraction. Purified microglia from two brains were pooled and used as one sample.

### 2.4. Laser Capture Microdissection of Mouse Nigral Neurons

Laser capture microdissection (LCM) of nigral neurons from brains of AT2 KO (*n* = 6) and the corresponding controls (*n* = 6) were performed as we previously described [37]. Briefly, mice were stunned with CO_2_ and then killed by decapitation. Then brains were rapidly removed, frozen in liquid nitrogen and stored at −80 °C. Then, tissue coronal sections (20 µm thick; Shandon Cryotome, ThermoFisher) containing the substantia nigra were cut with a cryostat, mounted on glass slides, and stained with neutral red (1%; 2 min on ice). After complete dehydration, LCM was performed using a PALM MicroBeam (Zeiss, Jena, Germany) system controlled by PALM Robo software (PALM RoboSoftware 4.2, Zeiss). Nigral neurons were visualized under bright-field microscopy at 40× magnification with the aid of an AxioCam Icc camera (Zeiss). Cell pools (1000 neurons per mouse) were cut and catapulted by laser pulses into an adhesive microtube cap (Zeiss). RNA extraction from isolated cells was performed with the RNeasy microkit (Qiagen, Hilden, Germany) protocol. We checked that this method preserves the RNA integrity (see [37] for details).

### 2.5. Microglial and Neuronal Cell Cultures

Rat primary microglial cultures were prepared as previously described [38] and maintained with DMEM supplemented with 10% fetal bovine serum, 1% of penicillin/streptomycin (Sigma) and 1% of amphotericin b (Sigma) at 37 °C in a humidified incubator (5% CO_2_ and 95% air). Briefly, 1–2-day old rats were subjected to cervical dislocation followed by decapitation, brains extracted, meninges removed, and brain tissue mechanically dissected and filtered. The resulting cell suspension was centrifuged for 10 min at 2000 rpm and the pellet was resuspended in medium and plated on a 75 cm^2^ culture flask. Once confluent (12–16 days), flasks were agitated for 4 h at 180 rpm, and microglia were collected by centrifugation and re-seeded in fresh culture medium onto 35 mm culture dishes.

BV2 murine microglial cells [39] were cultured in Dulbecco’s Modified Eagles Medium (DMEM) containing 10% fetal bovine serum (FBS), 1% of penicillin/streptomycin and 1% of amphotericin b. Dopaminergic N27 cell line derived from rat female mesencephalic tissue (SCC048; Millipore, Darmstadt, Germany) was cultured in RPMI complete medium supplemented with 10% FBS, 2 mM glutamine, and the antibiotic cocktail as above. Cell lines were maintained at 37 °C in a humidified incubator (5% CO_2_ and 95% air) in a 75 cm^2^ culture flask. Once cells became confluent were re-seeded onto culture dishes before starting the experiments.

### 2.6. Treatment of Cultures

Microglial cells were treated for 8 h with the recombinant (pro)renin protein (5 nM; ab123479, Abcam) in the presence of the AT1 antagonist losartan (3 µM; Sigma), which was added 30 min before the (pro)renin treatment to exclude any possible AngII-dependent effect. The ROCK inhibitor fasudil (0.2 µM; LC laboratories), rapamycin (100 nM; Sigma) or the inflammasome inhibitor *N*-[[(1,2,3,5,6,7-Hexahydro-s-indacen-4-yl)amino]carbonyl]-4-(1-hydroxy-1-methylethyl)-2-furansulfonamide], MCC (10 µM; InvivoGen) were added 30 min before (pro)renin treatment. After treatments, microglia were analyzed, and conditioned medium from microglia (MCM) was collected, sterile-filtered with 0.22 μm filters to remove cell debris and stored at −80 °C. For MTT experiments, the N27 cell line was maintained in the microglial-conditioned medium for 8 h [20,40,41].

### 2.7. Immunofluorescent Labeling

Double immunofluorescence labeling was performed to confirm the presence of PRR in microglial cells [18]. BV2 and primary microglial cultures grown on glass coverslips were fixed in 4% paraformaldehyde in Dulbecco’s phosphate-buffered saline (DPBS; pH 7.4) and incubated overnight with anti-PRR (1:50; ab40790, Abcam) and anti-Ox42 (1:100; AbD Serotec) primary antibodies. After rinsing with DPBS, the sections were incubated for 150 min with the Alexa-conjugated secondary antibodies (1:200; Molecular Probes; Invitrogen). Finally, cells were stained with Hoechst (10 μg/mL; Sigma), and mounted with Immumount (Thermo Fisher Scientific, Waltham, MA, USA). Immunolabeling was visualized with a confocal laser-scanning microscope (AOBS-SP5X; Leica Microsystems).

### 2.8. NADPH-Oxidase and ROCK Activities

Microglial cells were homogenized and centrifuged, and the supernatant was assayed for protein concentration using the Pierce BCA Protein Assay Kit (Thermo Fisher Scientific). The NADPH-oxidase activity was measured in 15 μg of extracted proteins in the presence of NADPH (10^−4^ mol/L; Sigma) and lucigenin (5 × 10^−6^ mol/L; Sigma) with an Infinite M200 multiwell plate reader (Tecan, Männedorf, Switzerland). As a control, no enzymatic activity was detected in the absence of NADPH.

ROCK activity was measured with a ROCK activity assay kit (Cell Biolabs Inc, San Diego, CA, USA) according to the manufacturer’s instructions. Cells were homogenized in lysis buffer (50 mM Tris-HCl pH7.5, 150 mM NaCl, 1 mM 2-glycerophosphate, 1% Triton X-100, 1 mM EDTA, 1 mM EGTA, 1 mM Na_3_VO_4_), then centrifuged, and the supernatant was assayed for protein concentration (BCA, Pierce). Equal amounts of protein (25 µg per well) were used. ROCK activity was assessed by measuring the absorbance at 450 nm using the plate reader Infinite M200 (Tecan) [24,42].

### 2.9. RNA Extraction and Real-Time Quantitative RT-PCR

Total ribonucleic acid (RNA) from the rat nigral region or cell cultures was extracted with Trizol (Thermo Fisher Scientific) according to the manufacturer’s instructions. The concentration of RNA was estimated using the Nanodrop Spectrophotometer (Thermo Fisher Scientific). Total RNA (1 µg) was reverse transcribed to complementary DNA (cDNA) with deoxynucleotide triphosphate (dNTP), random primers, and Moloney murine leukemia virus reverse transcriptase (MMLV, Invitrogen, Paisley, UK).

Real-time PCR experiments were performed with a QuantStudio3 platform (Applied Biosystems, Foster City, CA, USA). β-actin or GAPDH were used as a housekeeping gene and was amplified in parallel with the genes of interest. The data were evaluated by the delta–delta Ct method (2^−ΔΔCt^) where Ct is the cycle threshold. The expression of each gene was obtained relative to the housekeeping transcripts. PrimerBLAST software was used to design oligonucleotide primers (Table 1) [29].

### 2.10. Western Blot Analysis and Autophagy Assay

Tissue or cells were homogenized in Radio Immunoprecipitation Assay (RIPA) buffer containing protease inhibitor cocktail (P8340; Sigma) and phenylmethanesulfonyl fluoride (PMSF) (P7626; Sigma). Homogenates were centrifuged and protein concentrations were determined with the Pierce BCA Protein Assay Kit. Equal amounts of protein were separated by 5–10% Bis-Tris polyacrylamide gel and transferred to the nitrocellulose membrane. The membranes were incubated overnight with primary antibodies against PRR (ab40790, Abcam), ROCK (sc-398519, Santa Cruz Biotechnology) IL1β (sc-12742, Santa Cruz Biotechnology). The corresponding HRP (horseradish peroxidase) conjugated secondary antibodies used were all from Santa Cruz Biotechnology.

Immunoreactivity was detected with an Immun-Star HRP Chemiluminescent Kit (170-5044; BioRad) and imaged with a chemiluminescence detection system (Molecular Imager ChemiDoc XRS System, BioRad). Blots were reprobed for HRP conjugated anti-tubulin (Abcam) as a loading control. Protein expression was measured by densitometry of the corresponding band and expressed relative to the tubulin band value. The data were then normalized to the values of the control group of the same batch (100%) to counteract possible variability among batches.

Autophagy was assessed by Western blot with an antibody detecting microtubule-associated protein 1 light chain 3 beta (LC3B). During autophagy soluble LC3B-I is converted to LC3B-II that is associated with autophagosomes. Detection of the autophagosome-specific form LC3B-II is widely used to monitor autophagy.

Cell homogenization and protein determination were carried out as described above. Equal amounts of protein were dissolved in RIPA containing DTT (0.01 M), separated by 5–14% Bis-Tris polyacrylamide gel and transferred to PVDF membranes. The membranes were incubated overnight with a primary antibody against LC3B-II (1:1000; 2775, Cell signaling). Finally, protein expression was analyzed as described above [29,43].

### 2.11. MTT Viability Assay

The 3-(4, 5-dimethylthiazolyl-2)-2, 5-diphenyltetrazolium bromide (MTT) assay was performed to measure cell viability. N27 dopaminergic neurons were plated in multiwell plates for 24 h before treatments. Cells were treated or not treated with the dopaminergic neurotoxin 6-OHDA (40 µM in 0.02% saline ascorbate) to induce a slight decrease in viability. The cultures were grown in conditioned medium (CM) from microglial cultures untreated or treated with PR or PR plus the ROCK inhibitor fasudil. MTT solution (20 µL/well, 5 mg/mL in PBS; Sigma) was added to each well and cells were incubated for 4 h at 37 °C. Culture supernatant was then carefully removed and the formazan crystals resulting from cellular dehydrogenases were dissolved in acidic isopropanol and quantified at 570 nm using the plate reader Infinite M200 (Tecan). The absorbances were expressed as a percentage of control [44].

### 2.12. Statistical Analysis

All data were obtained from at least three independent experiments and expressed as mean values ± SEM. Two group comparisons were performed with Student’s t-test. Multiple comparisons were analyzed with a one-way analysis of variance followed by the Holm–Sidak post-hoc test. The test used for each experiment is specified in the corresponding figure legend. The normality of populations and homogeneity of variances were tested before each analysis of variance. Differences at *p* < 0.05 were considered statistically significant. Statistical analyses were carried out with SigmaStat 3.0 from Jandel Scientific.

## 3. Results

### 3.1. Upregulation of (Pro)Renin Receptors in Nigral Inflammatory Processes. Upregulation in Microglia

We initially investigated the possible involvement of PRR in nigral inflammatory processes using several models of rats and mice that had been previously shown to have upregulation of pro-inflammatory markers in the nigral region [15,29,31,32].

**Figure 2:**
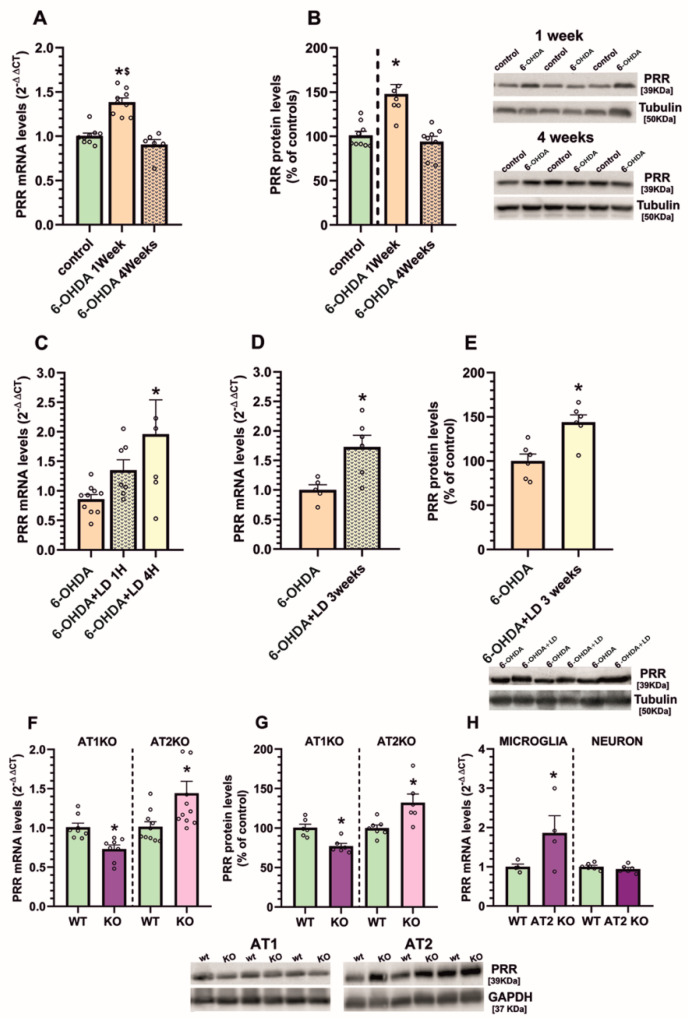
mRNA and protein expression of (pro)renin receptors (PRR) in the nigral region of several rat and mouse models. In comparison with control (i.e., unlesioned) rats, we observed upregulation in PRR expression one week after 6-hydroxydopamine injection, which had decreased to control levels 4 weeks after lesion (**A**,**B**). However, PRR expression increased again in long-term lesioned rats after a single (**C**) or daily injection (for 3 weeks; **D**,**E**) of L-DOPA, together with the dyskinetic response. PRR expression was increased in AT2 KO mice and decreased in AT1 KO mice relative to controls (**F**,**G**). Analysis of microglia and neurons isolated from AT2 KO mice detected an increase in PRR expression in microglia (**H**). Protein expression was measured relative to the tubulin or GAPDH band value and mRNA expression was measured relative to that of the housekeeping transcripts (β-actin). For mRNA, the comparative cycle threshold values method (2^−ΔΔCt^) was used. Protein values were normalized to the values for controls (100%). Data are mean ± SEM. * *p* < 0.05 relative to the corresponding control, $ *p* < 0.05 compared to 4-week group. Student’s t-test and One-way ANOVA followed by Holm Sidak post hoc test (**A**–**C**). 6-OHDA, 6-hydroxydopamine; AT1, angiotensin type 1 receptor; AT2, angiotensin type 2 receptor; KO, knock out, LD, Levodopa; mRNA, messenger ribonucleic acid; PRR, (pro) renin receptor.

First, we used the classical 6-OHDA PD model. One week after injection of 6-OHDA in the MFB (i.e., in the acute period of nigral neuroinflammation and neurodegeneration) we observed significant upregulation of PRR mRNA and protein in the nigral region, which had been downregulated to control levels 4 weeks after the 6-OHDA injection (i.e., when the degenerative and inflammatory process is already stabilized) (Figure 2A,B). In these *nigras* with long-term stabilized lesions, L-DOPA injection upregulates again the neuroinflammatory markers, which is involved in the development of L-DOPA-induced dyskinetic movements (see 29 for details). In this model, we observed that PRR was upregulated 1h and, particularly, 4 h after a single L-DOPA injection (Figure 2C). In a more chronic model of dyskinesia obtained by daily injection of L-DOPA, PRR was upregulated 90 min after the last injection, as previously observed for other pro-inflammatory markers [29] (Figure 2D,E).

Then, we checked levels of PRR in mice deficient for other RAS receptors and that have previously shown downregulation (AT1 KO mice) or upregulation (AT2 KO mice) of pro-inflammatory markers. Both at mRNA and protein levels, AT1 KO mice showed downregulated PRR expression and AT2 KO mice showed upregulation of PRR expression (Figure 2F,G).

Finally, we used this last model (KO AT2 mice) to know if this neuro-inflammation-related increase in PRR occurs in microglial cells or in neurons (Figure 2H). Microglia isolated from brains of adult KO AT2 mice showed increased expression of PRR mRNA relative to microglia isolated from brains of adult control mice. However, neurons isolated from the nigra of adult KO AT2 brains using laser microdissection did not show any significant upregulation of PRR mRNA expression.

### 3.2. Cultured Microglia Express (Pro)Renin Receptors That Activated by (Pro)Renin Upregulate Major Components of the Microglial Pro-Inflammatory Response

**Figure 3:**
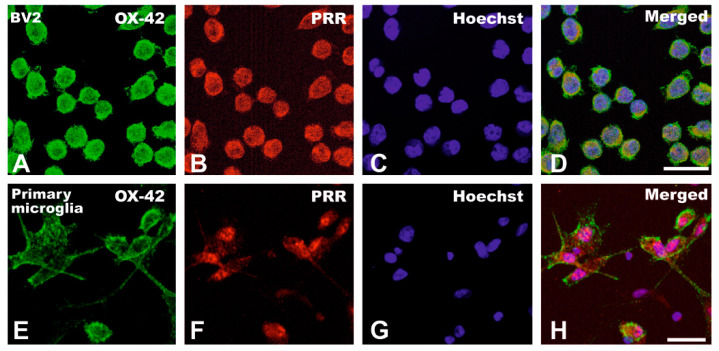
Double immunolabeling for the microglial marker OX-42 (green) and PRR (red) in BV2 microglial cells (**A**–**D**) and primary microglia (**E**–**H**). Scale bars for (**A**–**H**): 50 µm. PRR, (pro) renin receptor.

We initially confirmed by immunohistochemistry the presence of PRR in the microglial cell line BV2 and in primary microglia, which showed clear double labeling for PRR and the microglial marker OX-42 (Figure 3).

**Figure 4:**
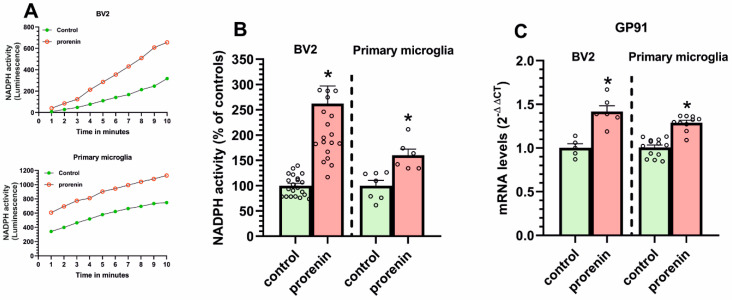
NADPH-oxidase activity (**A**,**B**) and the NADPH-oxidase gp91 subunit expression (**C**) in BV2 and primary microglia treated or untreated (control) with recombinant prorenin (5 nM) in the presence of losartan (100 nM). The time-course of a single experiment (0–10 min) is shown in (**A**), and the accumulative value from all experiments at 10 min is shown in (**B**). The mRNA expression was determined relative to the housekeeping transcripts (β-Actin). For mRNA, the comparative cycle threshold values method (2^−ΔΔCt^) was used. Data are mean ± SEMs. * *p* < 0.05 relative to the corresponding control (Student’s *t*-test). NADPH oxidase, nicotinamide adenine dinucleotide phosphate oxidase.

In the presence of the AT1 receptor blocker losartan, to exclude any effect via AT1 receptors, we analyzed the effect of treatment with PR on the microglial NADPH-oxidase complex activity (Figure 4A,B). Both in BV2 and primary microglia, we observed a PR-induced increase in NADPH-activity. Consistent with this, we observed that PR treatment induced an increase in the mRNA expression of the membrane NADPH-oxidase complex subunit gp91 (Figure 4C).

**Figure 5:**
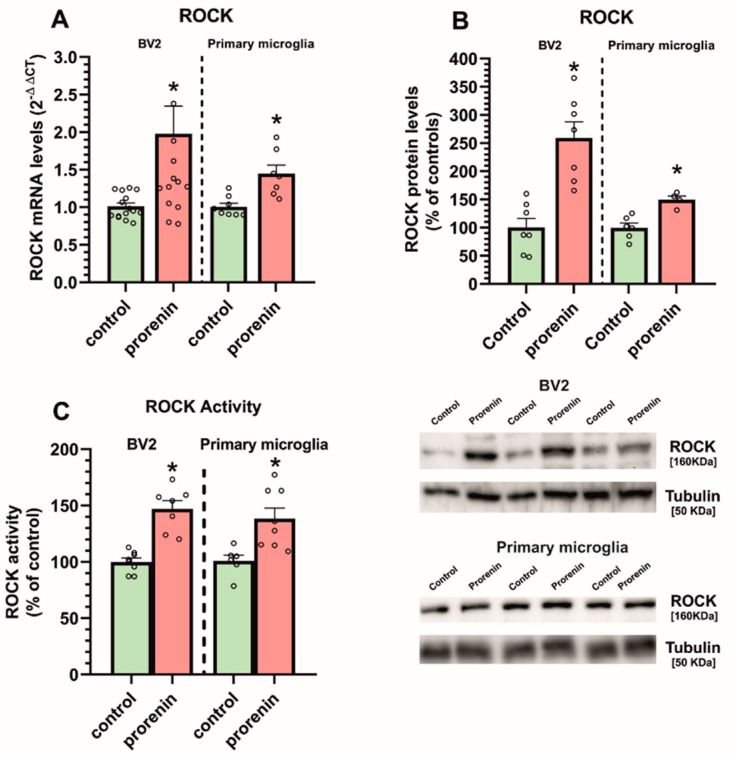
ROCK mRNA and protein expression (**A**,**B**) and ROCK activity (**C**) and in BV2 and primary microglia treated or untreated (control) with recombinant prorenin (5 nM) in the presence of losartan (100 nM). The mRNA expression was determined relative to the housekeeping transcripts (β-Actin). For mRNA, the comparative cycle threshold values method (2^−ΔΔCt^) was used. Data are mean ± SEMs. * *p* < 0.05 relative to the corresponding control (Student’s *t*-test). ROCK, Rho-kinase.

A second major component involved in promoting the pro-inflammatory microglial response is the RhoA/ROCK pathway. In the presence of losartan, we treated microglial cultures with PR and determined ROCK expression, which was significantly increased both at mRNA and protein levels in BV2 and primary microglia (Figure 5A,B), together with a significant increase in ROCK enzymatic activity (Figure 5C).

**Figure 6:**
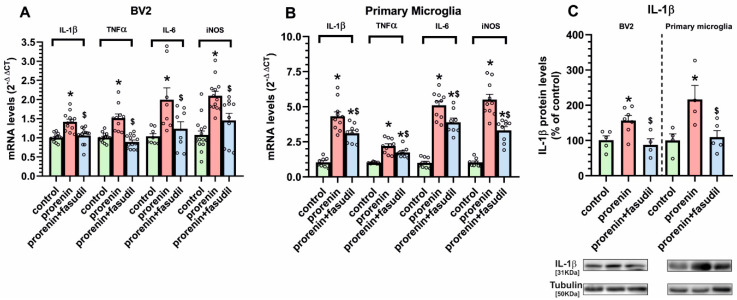
IL-1β, TNF-α, IL-6 and iNOS mRNA expression (**A**,**B**) and IL-1β protein expression (**C**) in BV2 and primary microglia treated or untreated (control) with recombinant prorenin (5 nM) or prorenin plus the ROCK inhibitor fasudil (0.2 µg/µL) in the presence of losartan (100 nM). Protein expression was measured relative to the tubulin band value and mRNA expression was measured relative to that of the housekeeping transcripts (β-Actin). For mRNA, the comparative cycle threshold values method (2^−ΔΔCt^) was used. Protein values were normalized to the values for controls (100%). Data are mean ± SEM. * *p* < 0.05 relative to the corresponding control, $ *p* < 0.05 relative to the prorenin-treated group. Student’s t-test and One-way ANOVA followed by Holm Sidak post hoc test. IL-1β, interleukin 1β; IL-6, interleukin 6; iNOS, inducible nitric oxide synthase; mRNA, messenger ribonucleic acid; TNF-α, tumor necrosis factor-alpha.

Consistent with the above-mentioned findings, we observed that, in the presence of losartan, PR induced a significant increase in the expression of major markers of the pro-inflammatory microglial response (IL-1β, TNF-α, IL-6, iNOS) both in BV2 and primary microglia (Figure 6A,B). Interestingly, the PR-induced increase in microglial pro-inflammatory markers was inhibited by simultaneous treatment with the ROCK inhibitor fasudil, which confirms the major role of ROCK activation in the PR-induced microglial pro-inflammatory response.

### 3.3. Microglial Autophagy and Inflammasome Are Involved in the (Pro)Renin-Induced Microglial Pro-Inflammatory Response

**Figure 7:**
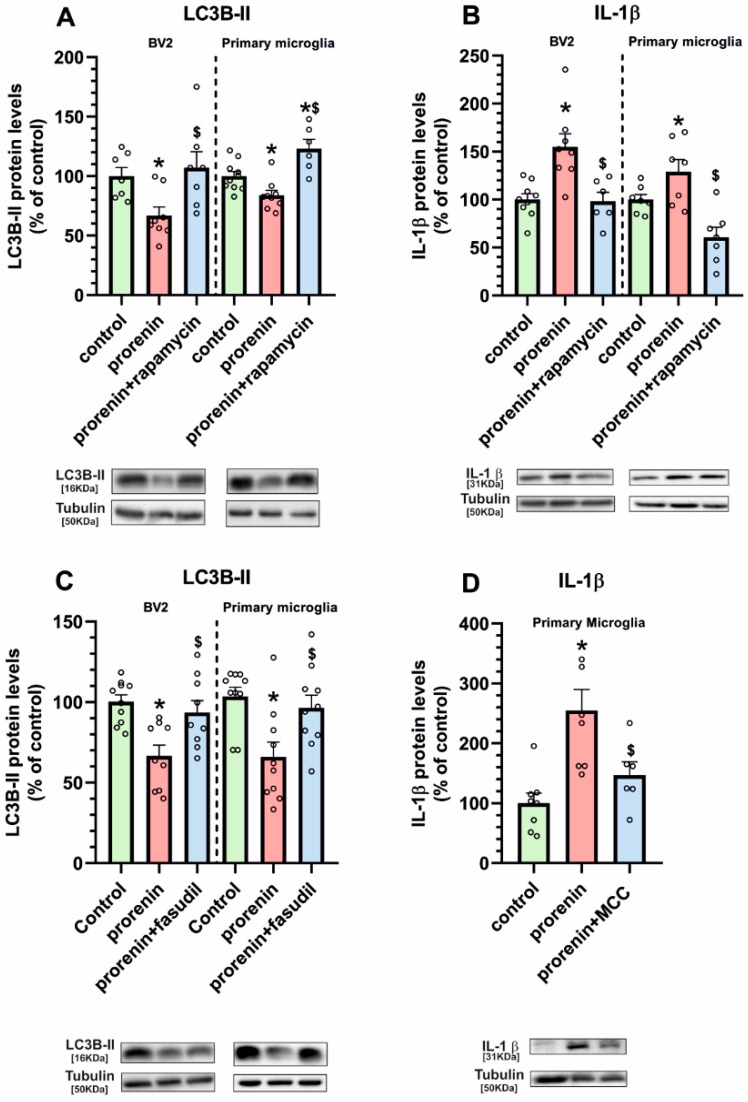
LC3B-II (**A**,**C**) and IL-1β (**B**,**D**) protein expression in BV2 and primary microglia treated or untreated (control) with recombinant prorenin (5 nM) or prorenin and the autophagy inducer rapamycin (1 M) (**A**,**B**) or prorenin and the ROCK inhibitor fasudil (0.2 µg/µL) (**C**) or prorenin and the inflammasome inhibitor MCC (10 µM) (**D**), in the presence of losartan (100 nM). Protein expression was measured relative to the tubulin band value and normalized to the values for controls (100%). Data are mean ± SEM. * *p* < 0.05 relative to the corresponding control, $ *p* < 0.05 relative to the PR-treated group (One-way ANOVA followed by Holm Sidak post hoc test). IL-1β, interleukin 1β; LC3B, microtubule-associated protein 1 light chain 3 beta; MCC, *N*-[[(1,2,3,5,6,7-Hexahydro-s-indacen-4-yl)amino]carbonyl]-4-(1-hydroxy-1-methylethyl)-2-furansulfonamide]; ROCK, Rho-kinase.

Treatment of BV2 and primary microglial cells with PR, in the presence of losartan, induced a significant reduction in levels of the autophagy marker LC3B-II, which were restored by simultaneous treatment with the autophagy inducer rapamycin (Figure 7A). To confirm that this effect is involved in the PR-induced pro-inflammatory microglial response, we studied the effect of PR treatment on IL-1 β expression in the presence of losartan and the autophagy inducer rapamycin in BV2 and primary microglial cultures. As in previous experiments, PR induced a significant increase in microglial levels of IL-1 β, which was inhibited by simultaneous treatment with rapamycin (Figure 7B). Interestingly the PR-induced decrease in the autophagy marker LC3B-II was also inhibited by simultaneous treatment with the ROCK inhibitor fasudil, revealing the major role of ROCK activation in the PR-induced decrease in autophagy (Figure 7C).

Activation of the NLRP3 inflammasome complex plays a major role in the production of pro-inflammatory cytokines such as IL-1 β. The present results suggest that activation of the inflammasome is also involved in the pro-inflammatory effects induced by PR in microglia, as we observed that PR-induced IL-1 β upregulation was inhibited by the inflammasome inhibitor MCC (Figure 7D).

### 3.4. PRR Activation Significantly Increases the Deleterious Effect of Microglia on Dopaminergic Neurons

**Figure 8:**
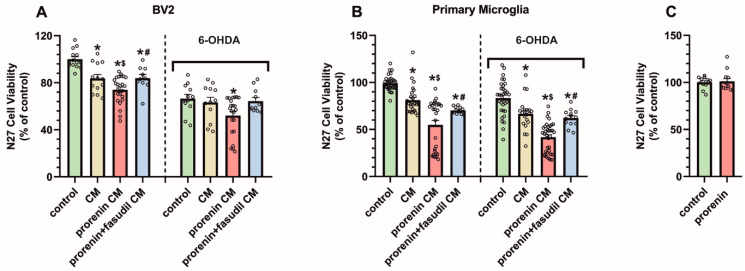
N27 dopaminergic cell viability in cultures, treated or untreated (controls) with low doses of the neurotoxin 6-hydroxydopamine, and conditioned medium (CM) from BV2 (**A**) or primary (**B**) microglial cultures, or CM plus recombinant prorenin (5 nM), or CM plus prorenin plus the ROCK inhibitor fasudil (0.2 µg/µL) (in the presence of losartan; 100 nM). N27 cells were also treated with culture medium containing the same doses of prorenin and losartan (in the absence of microglia), and no significant effect on N27 cell viability was observed (**C**). Data were normalized to the values for untreated controls (100%). Data are mean ± SEM. * *p* < 0.05 relative to the corresponding control, $ *p* < 0.05 relative to the CM-treated group, # *p* < 0.05 relative to the CM + prorenin-treated group (One-way ANOVA followed by Holm Sidak post hoc test in A, B; Student’s t-test in C). 6-OHDA, 6-hydroxydopamine; CM, conditioned medium.

We cultured N27 dopaminergic neurons in the presence of BV2 or primary microglia conditioned medium, from microglial cultures treated or not treated with PR, and in the presence or absence of a low doses of the dopaminergic neurotoxin 6-OHDA (Figure 8A,B). Administration of conditioned medium from PR-treated microglial cultures led to a significant decrease in dopaminergic cell viability relative to conditioned medium from microglial cultures not treated with PR. This was observed both in N27 dopaminergic cell cultures treated or untreated with low doses of 6-OHDA. Consistent with the above-mentioned results, the enhancing effect on dopaminergic cell death induced by conditioned medium from PR-treated microglia was inhibited by simultaneous treatment of microglia with PR and fasudil. Finally, we treated N27 cells with culture medium containing PR and losartan (i.e., without microglia) to exclude any possible direct effect of PR on N27 cells, and we did not observe any significant effect on N27 cell death (Figure 8C).

## 4. Discussion

The major observations of the present study are that the PRR is upregulated in the nigral region, particularly in microglia, in animal models showing an inflammatory or pro-inflammatory state. Furthermore, treatment of microglial cells with PR, in the presence of the AT1 receptor blocker losartan, induces the expression of microglial pro-inflammatory markers, which is mediated by upregulation of NADPH-oxidase and ROCK activities, and downregulation of autophagy and upregulation of inflammasome activity are also involved. Interestingly, conditioned medium from PR-treated microglia increased dopaminergic cell death relative to medium from non-treated microglia. However, the effects of conditioned medium from PR-treated microglia on dopaminergic cell death were blocked by simultaneous treatment of microglia with the ROCK inhibitor fasudil, which denotes the major role of ROCK activation in the PR pro-inflammatory effects.

We first used a series of rodent models, in which we and other research groups previously showed upregulation of pro-inflammatory markers in the nigral area, to check levels of PRR expression. In short-term 6-OHDA lesions [15,29] and AT2 KO mice [32], we observed an upregulation of PRR expression, which decreased to control levels several weeks after 6-OHDA lesions (i.e., when neuroinflammatory markers decreased) [15,29]. However, PRR was below control levels in mice characterized by downregulation in the neuroinflammatory responses such as AT1 KO mice [30,31], and was increased again in long-term 6-OHDA lesions treated with L-DOPA, which induces dyskinesia and upregulation in inflammatory markers [27,28,29]. The results were consistent with previous studies in several peripheral tissues showing pro-oxidative and pro-inflammatory effects after PRR activation [45,46,47]. In the brain, PRR was observed in neurons and microglial cells [17,18,19], and at low levels [18,19] or undetectable [17,48,49] in astrocytes. To investigate if the increase in PRR expression in the pro-inflammatory state was related to neurons or microglial cells, we isolated microglial cells, using anti-CD11 microbeads and neurons, using laser microdissection, from brains of adult AT2 KO mice, and we detected a significant increase in PRR expression in microglial cells but not in neurons.

Then, we used cultures of BV2 microglial cells and primary microglial cells to study the effects of PR-induced PRR activation on major pathways involved in pro-inflammatory microglial responses. In these experiments, we blocked AT1 receptors with losartan to exclude any possible indirect effect of PR related to the increase in levels of AngII by hydrolysis of angiotensinogen. We first observed an increase in NADPH-oxidase activity, which is known to play a major role in the microglial-pro-inflammatory response induced by other stimuli, including activation of AT1 receptors [15,16,24]. Activation of the NADPH-oxidase complex induces superoxide production, which is released in large proportions to the extracellular space but also triggers different intracellular pathways involved in the inflammatory response, including activation of ROCK [24,50].

The results suggest that the RhoA/ROCK pathway also mediates the pro-inflammatory effects of microglial PRR stimulation. The PR-induced increase in microglial pro-inflammatory markers was inhibited by treatment with the ROCK inhibitor fasudil. ROCK activation has been shown to play a major role in the microglial pro-inflammatory response, including migration of microglia into the inflamed areas [24,51,52,53]. Our research group and others have shown that ROCK inhibitors have neuroprotective effects against dopaminergic degeneration in PD models [42,54,55], and that inhibition of the microglial pro-inflammatory response plays a major role in the neuroprotective effect [23,42,56,57]. More recently, we showed that the ROCK inhibitor fasudil inhibits the dyskinetic behavior induced by L-DOPA in PD models, which is mediated by inhibition of the neuroinflammatory response [29]. The present study suggests that fasudil-mediated inhibition of the PR-induced microglial pro-inflammatory response is involved in those protective effects.

The autophagic process has also been involved in the microglial pro-inflammatory response, as the autophagy inducer rapamycin decreased levels of pro-inflammatory markers in microglial cell lines [58,59]. In the present study, we observed that PR treatment significantly decreased autophagy levels (LC3B-II) in BV2 and primary microglia, which was reversed by rapamycin, suggesting the involvement of autophagy inhibition in the observed effects. Consistent with the above-mentioned studies, the PR-induced increase in IL-1β was reverted by simultaneous treatment with rapamycin. Interestingly, the PR-induced decrease in the autophagy marker was also inhibited by the ROCK inhibitor fasudil, revealing that ROCK activation is involved in the PR-induced reduction in autophagy and subsequent effects on microglial pro-inflammatory markers. This is consistent with previous observations in neurons showing that ROCK inhibition increases autophagic flux [60] and reduces levels of neuronal protein aggregates in several models [61,62,63]. Although effects of PR on microglial autophagy had not previously been observed, an important interaction between PRR and autophagy has been observed in some peripheral cells such as podocytes or cardiomyocytes [43,64]. In those cells, PRR plays a major role in autophagy as PRR inactivation inhibited the autophagic process leading to protein aggregates accumulation. Note, however, that PRR is a multifunctional protein and in those cells, this role was played by a truncated PRR form, which cannot bind PR, but plays a major role in the autophagic process [10,65]. The present experiments show that PRR activation by its specific ligand PR induces a decrease in autophagy in microglia and an increase in the pro-inflammatory response. The inflammasome complex is involved in the production of pro-inflammatory cytokines such as IL-1β or IL-18 [66,67]. Consistent with this, we observed that PR-induced increase in IL-1 β levels is inhibited by simultaneous treatment with the inflammasome inhibitor MCC, suggesting the involvement of inflammasome complex in the effects of PRR activation. Our results are also consistent with observations showing that autophagy may inhibit inflammasome-related responses by degrading inflammasome components and cytokine precursors [68].

Finally, we used cultures of the N27 dopaminergic cell line to know whether the above-described pro-inflammatory effects of PRR activation may have a significant effect on the well-known deleterious effect of the microglial pro-inflammatory response on dopaminergic neuron death [69,70]. As microglial effects can be mimicked by microglial conditioned medium, N27 dopaminergic neurons, untreated or treated with low doses of 6-OHDA, were grown in microglial conditioned medium, and we observed that culture medium from PR-treated microglia increased the dopaminergic cell death relative to culture medium from untreated microglial cultures, confirming the functional relevance of the PRR-derived pathways in the pro-inflammatory microglial response. We also treated N27 cells with culture medium containing PR and losartan (i.e., without microglia) to exclude any possible direct effect of PR on N27 cells, and we did not observe any significant effect on N27 cell death, confirming the major role of PR-induced activation of microglia in the deleterious effects on dopaminergic cell death observed in the present study. In addition, the deleterious effects of culture medium from PR-treated microglia on dopaminergic cell death were inhibited by simultaneous treatment of microglia with fasudil, confirming the major role of microglial ROCK activation in the pro-inflammatory effects induced by PRR activation, which is consistent with our previous studies on the effects of microglial ROCK inhibition on dopaminergic neuroprotection [23].

## 5. Conclusions

Activation of microglial PRR receptors enhances the microglial pro-inflammatory response and deleterious effects of microglia on dopaminergic cells, and microglial NADPH-oxidase, Rho-Kinase and autophagy are involved in this process. Microglial activation and neuroinflammation are involved in the progression of a number of brain diseases, and particularly the progression of neurodegenerative diseases, including Parkinson’s disease. Modulation of the microglial response and neuroinflammation appears as a major neuroprotective strategy, and the present results suggest that the inhibition of microglial PRR activity should be considered for neuroprotection.

## Figures and Tables

**Table 1:** Primer sequences.

	Gen	Forward Sequence (5′-3′)	Reverse Sequence (5′-3′)
**Rat/Mouse**	**Actβ**	TCGTGCGTGACATTAAAGAG	TGCCACAGGATTCCATACC
**Rat**	**Gp91**	ATCTTGCTGCCAGTGTGTCG	AATGGTGTGAATGGCCGTGTG
	**IL-1β**	GGCAACTGTCCCTGAACTCA	TGTCGAGATGCTGCTGTGAGA
	**IL-6**	GGATACCACCCACAACAGACC	AGTGCATCATCGCGTTCATACACA
	**iNOS**	CAGGCTTGGGTCTTGTTAGCC	GCCATGTCTGTGACTTTGTGCTT
	**PRR**	TGGTGGGAATGCAGTGGTAGAG	GGGACTTTGGGTGTTCTCTTGTT
	**ROCKII**	GTTCAGTTGGTTCGTCATAAGGCA	TGAACCACCCACGGACTGTT
	**Tnf-α**	CACGTCGTAGCAAACCACCA	GGTTGTCTTTGAGATCCATGCCA
**Mouse**	**Gp91**	GGAGTTCCAAGATGCCTGGA	CCACTAACATCACCACCTCATAGC
	**IL-1β**	GCTATGGCAACTGTTCCTGA	TGATGTGCTGCTGCGAGA
	**IL-6**	GACTGATGCTGGTGACAAC	GAGTGGTATCCTCTGTGAA
	**iNOS**	TGGTGAAGGGACTGAGCTGTTA	CAGGGGCAAGCCATGTCTGAG
	**PRR**	GTTTGTTGTCTCGTCATAAGC	ACTCTACCACTGCGTTCC
	**ROCKII**	CGAATAGAACTCCAGATGACC	GCACAGGCAATGACAACC
	**Tnf-α**	TGTGCTCAGAGCTTTCAACAA	CTTGATGGTGGTGCATGAGA

## Data Availability

Data is contained within the article and supplementary material.

## Supplementary Materials

The following are available online at https://www.mdpi.com/article/10.3390/antiox10091340/s1, Supplementary Methods and Figure S1: Amphetamine-induced rotational behavior, cylinder test and development of dyskinesia in 6-hydroxydopamine lesioned animals.

## Abbreviations

6-OHDA	6-hydroxydopamine
ACE	angiotensin converting enzyme
ACE2	angiotensin converting enzyme 2
Ang	angiotensin
AT1	angiotensin type 1 receptors
AT2	angiotensin type 2 receptors
CM	conditioned medium
DMEM	Dulbecco’s modified Eagles medium
dNTP	deoxynucleotide triphosphate
DPBS	Dulbecco’s phosphate buffered saline
HRP	horseradish peroxidase
IL-1β	interleukin1β
IL-6	interleukin 6
iNOS	inducible nitric oxide synthases
LC3B	microtubule associated protein 1 light chain 3 beta
LCM	laser capture microdissection
LD	levodopa
MasR	Mas receptors
MCC	*N*-[[(1,2,3,5,6,7-Hexahydro-s-indacen-4-yl)amino]carbonyl]-4-(1-hydroxy-1-methylethyl)-2-furansulfonamide]
MCM	conditioned medium from microglia
MMLV	Moloney murine leukemia virus reverse transcriptase
MTT	3-(45-dimethylthiazolyl-2)-2, 5-diphenyltetrazolium bromide
mRNA	messenger ribonucleic acid
NADPH oxidase	nicotinamide adenine dinucleotide phosphate oxidase
NLRP	NOD-like receptor family pyrin domain-containing 3
PD	Parkinson´s disease
PMSF	phenylmethanesulfonyl fluoride
PR	(pro)renin
PRR	(pro) renin receptor
RAS	renin–angiotensin system
RIPA	radio immunoprecipitation assay
ROCK	Rho-kinase
RPMI	Roswell Park Memorial Institute
RT-PCR	reverse transcriptase polymerase chain reaction
TNF-α	tumor necrosis factor alpha.
