# Supplementary material for: NADPH-Oxidase, Rho-Kinase and Autophagy Mediate the (Pro)renin-Induced Pro-Inflammatory Microglial Response and Enhancement of Dopaminergic Neuron Death
Source: Antioxidants (Basel). 2021 Aug 25;10(9):1340. doi: 10.3390/antiox10091340 (PMC8472832; doi:10.3390/antiox10091340)
Supplement: Supplementary file 1 [file antioxidants-10-01340-s001.zip › antioxidants-1303511-supplementary.pdf]

Supplementary Methods

1. 6-OHDA lesions of the dopaminergic system

Surgery was performed on rats deeply anesthetized with a mixture of ketamine (50 mg/kg)/medetomidine (0.4 mg/kg) and mounted in a stereotaxic frame (Kopf Instruments). Lesions were performed in the right medial forebrain bundle to reach a complete lesion of the nigrostriatal pathway. The animals were injected with 12 µg of 6-OHDA (to provide 8 µg of 6-OHDA free base; Sigma) in 4 µl of sterile saline containing 0.2 % ascorbic acid. Stereotaxic coordinates were taken from [1]. The stereotaxic coordinates were 3.7 mm posterior to bregma, 1.6 mm lateral to midline and 8.8 mm ventral to the skull at the midline, in the flat skull position. The tooth bar was set at -3.3 mm. The solution was injected using a 5-µl Hamilton syringe coupled to a motorized injector (Stoelting), at a rate of 0.5 µl/min; the cannula was left in situ 2 min after injection. Four weeks post-surgery the efficacy of the lesion was evaluated with the amphetamine rotation test and the cylinder test (see below).

2. Amphetamine-induced rotation

Amphetamine-induced rotation was performed 4 weeks after the 6-OHDA injection to evaluate the extent of the dopaminergic lesion. Turning behavior was recorded in an automated rotometer (Rota-count 8, Columbus Instruments). Right and left full body turns were recorded over 90 min after a 2.5 mg/kg intraperitoneal injection of D-amphetamine dissolved in saline. Animals rotating more than 6 net full turns per minute in the direction ipsilateral to the lesion were used in this study (i.e. those corresponding to more than 90% depletion of dopamine fiber terminals in the striatum) [2].

3. Cylinder test

The cylinder test was used to evaluate forelimb akinesia and the efficacy of the lesion. [3]. Rats were placed individually and videotaped in a transparent glass cylinder (20 cm diameter) with two mirrors to allow visualization from all directions and observation of the animals when they were turned away. The animals were then allowed to move freely in the cylinder and to explore the environment. An observer blinded to the treatment of the rats counted the number of weight-bearing touches made with each forelimb until a total of 20 touches. The data were expressed as percent of touches with the lesioned paw relative to total. A normal symmetric animal would thus receive a score of 50% (indicated as a dashed line in the corresponding figures, whereas lesions usually reduce performance of the impaired paw to less than 20% of total wall counts.

4. L-DOPA-induced dyskinesia

L-DOPA (6 mg/kg or 12 mg/kg) was administered daily to each rat as a subcutaneous injection for 3 weeks. L-DOPA was combined with the peripheral DOPA decarboxylase inhibitor benserazide (10 mg/kg) dissolved in saline. Abnormal involuntary movements (AIMs) were evaluated according to a rat dyskinesia scale, as we previously detailed [4], during the first, 3th, 5th, 9th, 11th, 15th, 18th and 21th L-DOPA injection. Briefly, the animals were placed in individual transparent plastic cages without bedding material and were scored every 20 min after the injection of L-DOPA and for the entire time course of dyskinesias. The abnormal involuntary movements were classified into four subtypes according to their topographic distribution as limb (purposeless movements of the contralateral forelimb), orolingual (empty jaw movements and contralateral tongue protrusion), axial (dystonic posturing or twisting movements of the neck and the upper

part of the body towards the side contralateral to the lesion) and locomotive movement (circling movements away from the lesioned side). The forelimb and orolingual dyskinesia are predominantly seen as hyperkinesia, while the axial dyskinesia is essentially a dystonic movement. Enhanced manifestations of normal behaviors, such as grooming, gnawing, rearing and sniffing were not included in the rating. The severity of each AIM subtype was assessed using scores from 0 to 4 (1: occasional, i.e. present < 50% of the time; 2: frequent, i.e. present >50% of the time; 3: continuous, but interrupted by strong sensory stimuli; 4: continuous, not interrupted by strong sensory stimuli). The data are expressed as integrated total AIMs scores, where the total AIMs is calculated by the sum of each AIM subtype (limb + orolingual + axial) scores multiplied by the interval of observation (x20 min) [5].

Supplementary references

1. Paxinos, G.; Watson C. The rat brain in stereotaxic coordinates. 7th Edition. Academic *Press, New York, NY*. **2013.**

2. Winkler, C.; Kirik, D.; Bjorklund, A.; Cenci, M.A. L-DOPA-induced dyskinesia in the intrastriatal 6-hydroxydopamine model of parkinson's disease: relation to motor and cellular parameters of nigrostriatal function. *Neurobiology of Disease*, **2002,** *10*, 165-186. https://doi: 10.1006/nbdi.2002.0499.

3. Schallert, T.; Tillerson, J.L. Intervention strategies for degeneration of dopamine neurons in parkinsonism: Optimising behavioural assessment of outcome. *Clifton, NJ: Humana Press*. **1999.**

4. Lopez-Lopez, A.; Labandeira, C.M.; Labandeira-Garcia, J.L.; Muñoz, A. Rho kinase inhibitor fasudil reduces l-DOPA-induced dyskinesia in a rat model of

Parkinson's disease. *Br. J. Pharmacol.* **2020**, *177*, 5622-5641. https://doi.org/10.1111/bph.15275.

5. Cenci, M.A.; Lee, C.S.; Bjorklund, A. L-DOPA-induced dyskinesia in the rat is associated with striatal overexpression of prodynorphin- and glutamic acid decarboxylasemRNA. *Eur. J. Neurosci*. **1998,** *10*, 2694-2706.

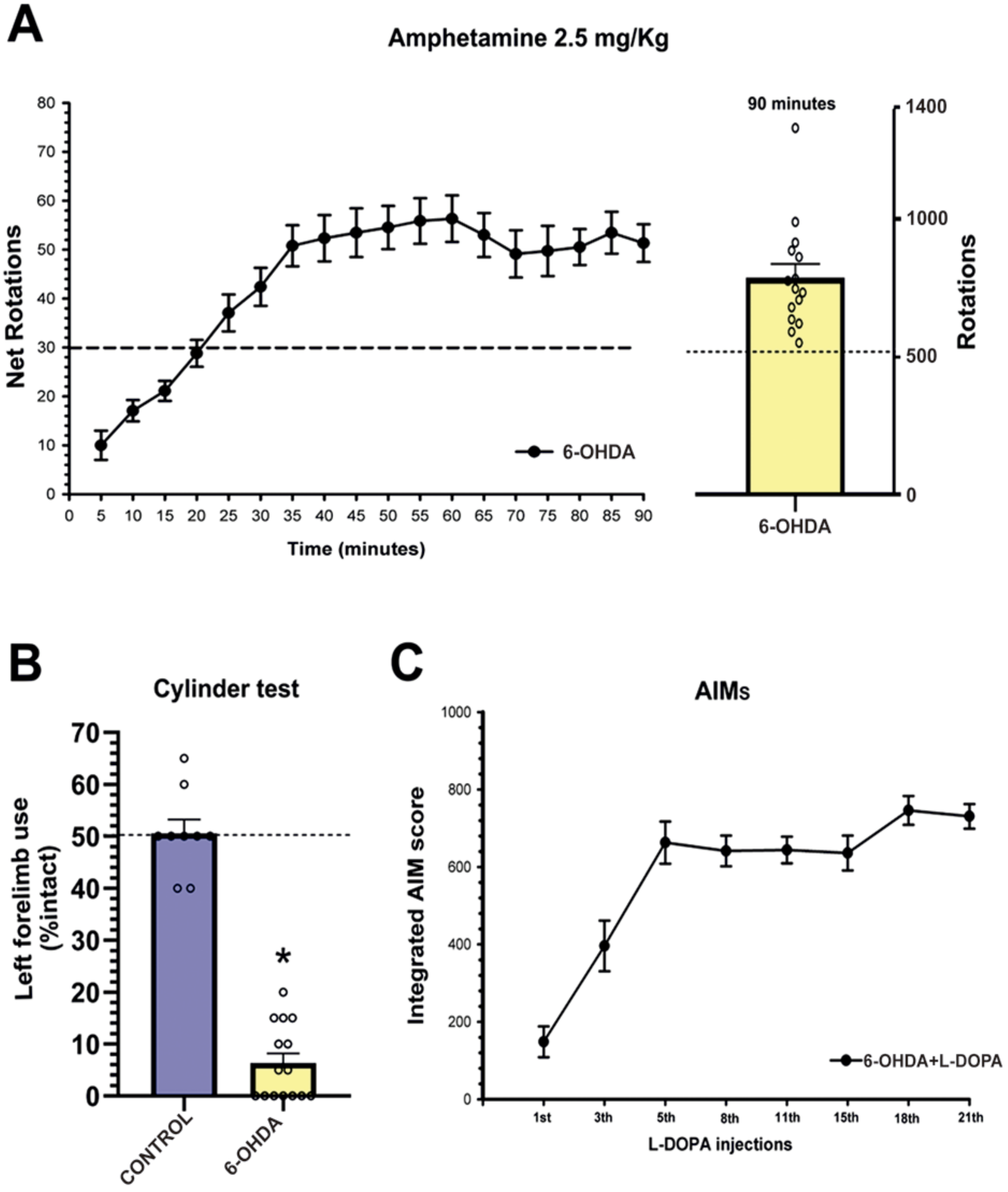

Figure S1. Amphetamine-induced rotational behavior, cylinder test and development of dyskinesia in 6-hydroxydopamine lesioned animals. **(A**) Time-course of amphetamine-induced rotation over a 90 min period, and accumulative rotational scores in the 90 min period. Animals showed an intense rotational behavior after amphetamine injection (787±50 turns in 90 min). (**B**) Cylinder test results showing a marked reduction in the left forelimb use relative to non-denervated controls. (**C**) Time-course of the effect of L-DOPA injections over the 21-day period, showing the dyskinetic behavior evaluated by the AIMs scale. Total AIMs score was estimated as the addition of limb, orolingual and axial components. AIMs, abnormal involuntary movements; 6-OHDA, 6-hydroxydopamine.
